# Autoethnography and cognitive adaptation: two powerful buffers against the negative consequences of workplace bullying and academic mobbing

**DOI:** 10.1080/17482631.2018.1459134

**Published:** 2018-04-18

**Authors:** Mpho M. Pheko

**Affiliations:** Department of Psychology, University of Botswana, Gaborone, Botswana

**Keywords:** Workplace bullying and mobbing, autoethnography, writing therapy, academic mobbing, meaning and sense making

## Abstract

Work is undoubtedly fundamental to human life, social development and the economic development of individuals, organizations and nations. However, the experience of working is not always pleasant as there are many instances where relationships between workers could deteriorate, leading to practices and behaviours that could be characterized as workplace bullying and/or mobbing. The current study is an exploratory study which used autoethnography to investigate experiences of academic bullying and mobbing, and relates the practices to power structures in academic institutions. Specifically, the author shares personal experiences and explores the physical and emotional pain of being bullied and mobbed. The author also outlines how both autoethnography and meaning in life strategies were used to cope with the physical and emotional distresses associated with the negative experiences. By outlining the success of the meaning in life strategies, the author hopes to inspire other “victims” to move from victims to being survivors of bullying and mobbing.

## Introduction

*Work is fundamental to the human condition … It allows us to engage with other people and it helps us to define our sense of identity. It provides us with access to the material necessities of life, as well as to the advantages and achievements of civilisation. Its allocation, organization, management and reward are therefore of no small importance*.(Abbot, 2006, p. 187)

Many scholars concur that organizational life, jobs and work are fundamental to the human condition, to human beings’ sense of identity (Abbot 2006; Du Gay, 1996), and are central to establishing personally meaningful self-definitions (Super, 1951; Meaning of Work [MOW], 1987). Even psychologists such as Freud (1930) identified work as a person’s strongest tie to reality. People work for a variety of reasons, including financial security, identity development, self-esteem, social acceptance, personal fulfilment, growth and/or generativity (Lemme, 2006). Work also plays a significant role in an adult’s life, such that our occupational role defines society’s expectations of us. Our work and jobs also serve as key determinants of our socioeconomic status and social class. Psychologists agree that the choice of an occupation is a major developmental task of young adulthood that reverberates throughout our lives (Lemme, 2006). All these are true whether work is performed for intrinsic or instrumental reasons (Abbott, 2006). It is therefore understandable how meaning of work research has long ranked work as relatively high in importance compared to other important life areas such as religion, leisure and the community (Harpaz, 1999; MOW, 1987).

While work is undoubtedly fundamental to human life, it is not always pleasant as there are many instances where relationships between workers could deteriorate, leading to hostile and hazardous behaviours, environments and outcomes (Chappell & Di Martino, 2006). It is important to note that not all violence is as overt as actual physical assault, verbal abuse or sexual harassment, as practices such as bullying, mobbing, harassment and intimidation are also considered violent practices (Jackson, Clare, & Mannix, 2002). Dysfunctional organizational behaviours have been related to varying organizational practices, such as managerial styles and approaches, organizational policies, processes, systems, strategic direction, organizational and reporting structures, interpersonal relationships, and organizational rules and regulations (Pheko, 2013; Pheko, Monteiro, & Segopolo, 2017). Many scholars and practitioners have shown interest in various factors that make work unpleasant. This paper specifically focuses on workplace bullying and mobbing, which have also been identified as a dysfunctional organizational behaviour.

### Aims, objectives and significance of the current study

The current study is an autoethnography investigating personal experiences of workplace bullying and mobbing, and outlines how writing therapy, cognitive adaptation and meaning in life strategies were used to survive the bullying and mobbing experiences. Specifically, the autoethnography presents a “personal story” as data, a research method and a therapeutic writing technique. Using an autoethnographic research approach was important because researchers have raised concerns that while research discusses both the psychological and physical costs of being bullied, the stories and conceptualizations of mistreatment by those targeted are not easily accessible (Akella, 2016; Tracy, Lutgen-Sandvik, & Alberts, 2006). The current autoethnography hopes to minimize this gap in research.

In similar work, Akella (2016) suggests that when using ethnography in cases of workplace bullying, it is advantageous to focus on one single case study or on one victim’s pain, humiliation and stress, as this will allow the reader to effectively deconstruct workplace bullying and classify it as an horrific and degrading process. The current study uses storytelling, and focuses on a single case study to articulate and explore the emotional pain and coping mechanisms used when going through workplace bullying and mobbing. It is important to note that more than one worker experienced the mobbing, and that their experiences are equally important and relevant. However, the autoethnography focuses mainly on my own experiences, feelings and actions, as they were easily accessible and verifiable to me. I chose this approach because I agree with those who think that the approach allows another person’s lived experiences to inspire others to reflect on their own experiences, and possibly to recontextualize their view of how the “other” has experienced life and culture (Bochner & Ellis, 1996). I also hope to possibly inspire others to move from victim to survivor of bullying and mobbing. Furthermore, I hope to encourage organizations to change their policies, processes and regulations to create environments that are less conducive for bullying and mobbing practices.

### Understanding the nature of workplace bullying and mobbing

Many but related definitions of workplace bullying and mobbing have been advanced, and scholars from different fields such as psychology, sociology, management, leadership and economics have studied workplace bullying and mobbing. Despite these differences in fields and the objectives of studies, and even theoretical and conceptual diversity, similar definitions and consequences of bullying and mobbing have emerged. The literature has consistently shown that actions and practices associated with bullying and mobbing can include withholding information, excluding the victim, social isolation, excessive criticizing or monitoring of the victim’s work, repeated negative acts, systematic mistreatment and victimization of targets, depriving the victim of work responsibilities, silent treatments, spreading rumours about the victim, attacking the victim’s private life, public humiliation, victimization, insulting remarks and even physical aggression (Einarsen, 1996, 1999; Einarsen, Hoel, Zapf, & Cooper, 2003; Einarsen & Skogstad, 1996; Hoel, Cooper, & Faragher, 2001; Salin, 2003).

In terms of outcomes, most victims of bullying and mobbing have been reported to experience feelings of inferiority in defending themselves, feelings of desperation and total helplessness, stress symptoms such disturbed sleep, recurring unpleasant nightmares, generalized anxiety disorder, difficulty falling asleep, moodiness, persistent symptoms of increased psychological arousal, incontinence, poor concentration, irritability, exaggerated startle responses, increased physiological reactivity when exposed to stimuli suggestive of the traumatizing problems and excessive feelings of guilt (Björkgvist, Osterman, & Hielt-Bdck, 1994; Einarsen, 1999; Einarsen & Skogstad, 1996; Randall, 1997). Using the Work Harassment Scale, Björkgvist et al. (1994) revealed experiences of insomnia, apathy, lack of concentration, post-traumatic stress disorder, social phobia, depression, anxiety and aggression.

In terms of conceptualization, researchers and practitioners have tended to use the terms “bullying”, “mobbing” and “harassment” interchangeably (Kircher, Stilwell, Talbot, & Chesborough, 2011). Sometimes, different cultural or geographical areas, and even countries, have used different terms such as mobbing, bullying or harassment to refer to the same problem, actions and practices (Di Martino, Hoel, & Cooper, 2003; Leymann, 1996). However, the two concepts of bullying and mobbing are different. Einarsen et al. (2003) state that mobbing is the term of choice in German-speaking countries, the Netherlands and some Mediterranean countries, while English-speaking countries have chosen to use the term bullying. They conclude that the two terms are used interchangeably to refer to the same phenomenon.

In differentiating between the two terms, Friedenberg (2008) defines mobbing as practices and behaviors carried out systematically and frequently over a long period by more than one co-worker against a colleague, and bullying as overt practices and behaviors by a single person—often a supervisor or more senior co-worker—against a vulnerable subordinate. While there are differences in conceptualization, harassment, bullying and mobbing can lead to similar consequences, such as a loss of dignity, lowered self-confidence and productivity, and an excessive amount of non-work-related stress and other related health issues (Kircher et al., 2011).

### Academic mobbing

Others have suggested a type of mobbing called “academic mobbing”, which happens mostly in institutions of higher learning. Academic mobbing has been defined as a non-violent and sophisticated kind of psychological bullying whereby one colleague is humiliated, intimidated, terrorized, ostracized and wrongly accused. This type of mobbing is important for the current paper because my story takes place in an academic setting. Pheko et al. (2017) presented an integrated conceptual framework explaining how certain organizational cultures and practices may motivate, facilitate, perpetuate, enable and precipitate workplace bullying. Supporting the conceptualization proposed by Pheko et al. (2017), researchers have suggested that academic culture in itself deserves to be given attention because the structure and practices of the academe can easily give birth to academic bullying and mobbing. Cassell (2011) explains that the conventional hierarchical structure of institutions of higher learning (i.e., president, chancellors, vice presidents, vice chancellors, deans of the various divisions and faculties, chairpersons of committees, and professors, lecturers and teaching assistants) creates a power structure that could make bullying and mobbing in the academe prevalent, significant and incessant.

For this paper, I have opted to use both terms (i.e., bullying and mobbing) because I was both bullied by my supervisor and targeted by more than one perpetrator, implying mobbing. Having noted the distinctions and similarities between workplace bullying, mobbing and academic mobbing, for the purpose of the current study I embraced the following definition of workplace bullying and mobbing: “harassment that inflicts a hostile work environment upon an employee by a coworker or coworkers, typically through a combination of repeated, inappropriate, and unwelcome verbal, nonverbal, and/or low-level physical behaviors that a reasonable person would find threatening, intimidating, harassing, humiliating, degrading, or offensive” (Von Bergen, Zavaletta, & Soper, 2006, p. 16).

## Methods and approaches

Using autoethnography as a research approach was an easy choice for the current study because I identified myself as both a subject and an object of the study. McIlveen (2008) explains that while qualitative data and methods have regained a legitimate place in psychological theory, research and practice, the story as data and as method is not yet comprehensively articulated within the field. As a methodology, autoethnography combines characteristics of autobiography and ethnography and uses research literature to analyse experiences, consider ways others may experience similar epiphanies and uses experience to illustrate facets of cultural experiences (Ellis, Adams, & Bochner, 2011). Chang, Ngunjiri, and Hernandez (2016) describe autoethnography as a study of the self, which entails writing about the experiences of life within the context of family, work, schooling and society, and also interpreting meanings and experiences. The objectives of autoethnographic research are achieved by presenting stories as data, as methods and as research approaches. An advantage of autoethnographic research is that instead of hiding or assuming that subjectivity, emotionality and the researcher’s influence on research should not exist, autoethnography acknowledges and accommodates emotionality and subjectivity (Ellis et al., 2011). Furthermore, the method permits the researchers to dig deeply into their own experience and attendant emotions in ways that may not be possible if they were being interviewed by someone else (Ngunjiri, Hernandez, & Chang, 2010).

### Understanding the autoethnographic research methodology

According to McIlveen (2008), autoethnography can align with both the constructivism–interpretivism and the critical ideological paradigms. McIlveen (2008) further explains that with respect to epistemology, autoethnographies relate to the notion of “lived experiences”, subjectivity and meaning within relative contexts, while from the perspective of ontology, autoethnographies assume “personal reality” to be a psychosocial construction. This makes the approach an excellent vehicle for researchers to come to understand themselves and others (Chang, 2016). Autoethnographies achieve the cultural understanding underlying autobiographical experiences by undergoing a similar qualitative research process which entails data collection, data analysis/interpretation and report writing (Chang, 2016). The approach as used in the current study had the following key features: (1) use of personal “accessible” writing; (2) “outing” the researcher’s experiences and shared humanity; and (3) embracing subjectivity (as suggested by Foster, McAllister, & O’Brien, 2006). Using this approach, uncertainty, presenting subjective perceptions and personal knowledge, was not only considered valid but also expected (Clandinin & Connelly, 1987).

### Procedure

Similar to the approach described by Ellis et al. (2011), in the current study patterns of the subject’s experiences were captured using tools such as formal letters, diaries, journal entries, memoirs and emails. First, the discovery that I was a target of bullying really shocked me and then later scared and worried me. But it also, somehow, empowered me. As soon as I confirmed that I was a target of bullying, I went back to the research literature and many other different sources to inform myself about the best ways of protecting myself and my career against this group of perpetrators. Going through the literature and different anti-bullying websites, I quickly learnt the importance of documenting the different actions, practices and behaviours of the perpetrators. The details were documented in what I called a “bullying and harassment log”, and the template included: (1) the name(s) of the perpetrator(s); (2) the dates and times when the practice or action took place; (3) why I believed that the practice and/or action constituted bulling or mobbing; (4) witnesses/bystanders present; and (5) action I took when the action or practice occurred. I also learnt early on that when dealing with workplace bullies, it is important to communicate officially, either through emails or through official letters. Therefore, the bullying and harassment log was used to inform emails to my supervisor, letters of complaint to the dean of the faculty and other official letters written to different offices at the university. The same bullying and harassment log, letters and emails later proved important when the matter was taken to the courts of law. The documentation also assisted me in establishing patterns and linkages that would have been difficult to establish without the log.

Ultimately, in terms of writing this autoethnography, the process followed the approach suggested by Chang (2016) and Ellis et al. (2011), which entailed engaging in the following reiterative process: (1) self-observation and examination of realities and experiences; (2) collection and verification of data (i.e., letters, emails, journal entries, bullying and harassment logs, as well as personal feelings and experiences; (3) reviewing the research literature to compare my experiences with others’ experiences, as well as to understand the meaning of events and the content being analysed; (4) reanalysing and interpreting data to decipher personal meanings of events, behaviours and thoughts; and (5) writing the autoethnography.

### Summary of “my story”

This summary should also be treated as a research approach and as the data that were analysed to produce the autoethnography. The story takes place in a large university, located in the capital city of a small African country called Botswana. I wish to start by noting that the type of bullying and mobbing I incurred could be profiled as supervisory bullying or academic mobbing (see subsection entitled academic mobbing) because all three of the primary perpetrators in my story had been the heads of the academic department at one point or another. Furthermore, all were still sitting on higher university committees where hiring, firing, promotion and compensation decisions were made. I was also aware, as it was public knowledge in the university, that the head of the institution was in a relationship with one of the perpetrators in my department. This relationship complicated the situation even further because my attempts to report the matter to the university higher offices and committees were met with contempt, which ultimately forced me to seek justice through the Botswana courts of law—a decision that almost bankrupted me emotionally, financially and physically. The head of the institution was later forced to resign from his position because of different allegations of maladministration.

My experience of being bullied began earlier than 2013; however, the critical incident and major events that led to the intensification of the bullying and mobbing actions and practices occurred in 2013. At that time, I had worked for the university for several years, completed my doctoral degree and had a few publications under my belt. Prior to this period I had never been verbally or formally warned for any form of indiscipline. My official performance records also showed that I was a diligent worker and a high performer, by all standards used. For many academics, and different academic institutions, quantity and quality of publications have been identified as the single most important criterion for tenure decisions, and the same applied to my employing university. With this understanding, a year before the bullying and mobbing practices intensified, six of my colleagues and I, who had noticed practices of unfairness in the department, decided to form a group to facilitate research and publication collaboratively. In 2013, looking purely at the standards and the university’s criteria for the appointment, promotion and review of academic staff at the university, a number of us qualified for promotion. Therefore, sometime in 2013, a colleague and I submitted our applications for promotion from the position of lecturer to senior lecturer. Having noted our efforts, the three senior staff members teamed together in a mob-like fashion and forged a plan to exclude, punish and humiliate the seven of us. We later learnt, through a secret report, that the three perpetrators had carefully designed and launched a plan to ruin our reputations and dismiss us from work, by manufacturing stories and relaying them to the higher offices of the institution. Fortunately or unfortunately, most of the other victims were on contracts; therefore, it was easy for their contracts to be terminated.

Unfortunately or fortunately for me, I had been hired as a permanent and pensionable staff member; therefore, the mob could not easily dispose of me. To fire me, they needed to be more creative. Because of this employment status, the three senior staff members carefully crafted well-planned propaganda which entailed writing secret reports and letters which contained fictitious incidents, incorrect statements, subjective evaluations, doctoring of minutes, professional character assassination and libellous insinuations, and presented them to the highest offices in the institution. Most of these letters were written and submitted in secret, and my supervisors falsely claimed that they had copied me in to the letters and other official documents. I only received most of the documentation when the university was forced to produce them by the courts of law. I noticed then that most of the reports had been collectively and carefully handpicked, nit-picked and selectively assembled to devalue my contribution to scholarship as well as to discredit me personally, all done with the intention of raising doubts among the promoting bodies regarding both my credibility and my abilities as a scholar.

Sometime in 2014, a couple of months after the bullying and mobbing intensified, I started experiencing a number of unexplained symptoms (which included but were not limited to insomnia, nightmares, stomach pains, heart palpitations, anxieties and extreme exhaustion, as well as intense neck and shoulder pain, excruciating headaches and dizziness). I would have consistent nightmares in which the three main perpetrators were the main actors. One night I woke up my husband, convinced that I had suffered a stroke because the entire right side of my body felt numb, and my fingers and toes on the same side of my body had started curling and contorting uncontrollably. We ended up at the hospital emergency room. After a number of days and a series of tests, a neurologist diagnosed me with complicated migraines; headaches which I continue to suffer three years layer.

After hospitalization, my colleagues advised me to start seeing a counsellor at the university’s counselling centre. With the assistance of the counsellor and colleagues, we established that I was a victim of workplace bullying and mobbing, a realization that shocked me. Prior to my experiences of being bullied, I had studied psychology—both general psychology and industrial–organizational psychology. I had also taught and had been present in many different settings (i.e., lectures, conferences and even social gatherings) where the topic of “bullying in the workplace” was discussed. However, and interestingly to me, it was only after sitting in front of a counsellor, after many months of bullying, that I was able to accept the diagnosis and label myself “a target of bullying”.

Throughout the period of bullying and mobbing, and before being suspended from work indefinitely, I went through varying and at times bizarre forms of mistreatment. For example, I was verbally assaulted and screamed at by one of the perpetrators in front of students. The perpetrator literally screamed profanity at me, just because I had asked her for the keys to a meeting room. I reported the matter to the head of department, who blatantly refused to act. I eventually gave up. When class allocations were done, I would not be allocated classes to teach, and when official meetings were called, my supervisors would intentionally not invite me. They would then later write to higher offices claiming that I had refused to teach classes and attend meetings. Another painful and humiliating incident entailed my head of department coming into one of my classes to inform me—in front of students—that I was getting kicked out of the class. Yet another incident involved my supervisor concocting a fictitious incident, claiming that his secretary had found me inside his office turning his files upside down. I later discovered through the same secretary that she never had such a conversation with the head of department: he had blatantly manufactured the story and passed it on to the human resources department as if it were true.

As the mobbing, bullying and harassment progressed, I followed the advice of colleagues and relatives and wrote a letter of complaint to the university, officially reporting and giving evidence of incidents of libel, fictitious claims, bullying, mobbing and harassment by the three perpetrators. A couple of days later, I guess in response, I was given a letter of disciplinary charges by the human resources director, who reports to the head of the university. The letter detailed some trumped-up charges—all assessed as serious and gross misconducts—with the possible consequence of summary dismissal, meaning that I would lose some of my hard-earned employment benefits. To make matters worse, the university refused to give me evidence of the charges, even when I persisted in asking to be provided with further and better particulars.

In the process of pushing the university to give me documents to substantiate their charges, I was given a report which was authored by the head of department and submitted to the highest offices in the institution. I was shocked because the head of department had only worked for the university and as my supervisor for around six months. The “report” was marred by fictitious incident after fictitious incident. For example, among many different accusations, my supervisor wrongfully informed the hiring bodies that I had published studies on albino rats which weighed some 270 kg—all done to defame me. The truth was that I had not conducted or had any work published on studies with rats. Nonetheless, this information was used against me, and rumours that I had published studies on big rats spread around the university like wildfire. The report also questioned the frequency of my publications, claiming that academics should only publish one or two articles per year. The truth was that working in a team with six of my colleagues had facilitated our research production as we had made a conscious effort to peer mentor and collaborate with each other. He also falsely alleged that that my publications did not investigate psychological variables. It is important to note that the perpetrators were the only psychologists sitting on the university committee responsible for promotion. Therefore, all the other committee members (who were non-psychologists) relied on their expertise when promotion and remuneration decisions were made. As the case progressed, the head of the institution used his power and authority to dissolve the sitting disciplinary committee, which had been generally fair towards the matter, and handpicked personal friends and associates to sit on a new committee that he formed. Colleagues alerted me to this and suggested that I resign from my job. I refused to resign because I knew that I was not guilty of any offence that I was being accused of.

Throughout these experiences, I felt like a criminal and kept asking myself: “I have worked for this university for years. My head of department only had six months’ tenure with the university. Before him, I have never had even one single verbal or written warning for any form of indiscipline. Why do all these seemingly smart people believe that I have started doing all these crazy things that my supervisor is claiming that I have done?”

Following the constitution of the new committee, I received yet another letter indicating that I was being suspended from work indefinitely because I was “under investigation”. The suspension letter indicated that I was not allowed to enter any building belonging to the institution—a public institution, for that matter. I felt like a criminal. My children wanted to know why I was not going to work, and I blatantly lied to them, claiming that I was on unpaid leave. Serving suspension was not enough for the mob. As I was being served with a suspension, the head of department created yet another fictitious story, claiming that my husband and I had come to the university to scream insults at him. Unfortunately for him, the date he chose was a public holiday in Botswana, meaning that no one was at work and that no one should have believed him. Yet, no action was taken against him for creating this fictitious incident. Around the same time, and given the development, I agreed to seek justice through the courts of law. Just before we appeared in court, the university changed the disciplinary charges by withdrawing some of the trumped-up charges and adding even more bizarre ones. Going to court was yet another horrible experience, as the anxieties, palpitations, headaches, nightmares and stomach problems returned every time I had go to court to listen to two lawyers revisiting the incidences and then having to wait for the judges to deliver judgments. I was successful in getting a final interdict at the first court—a decision that was later appealed by the university to the highest court in the land. After three terrible years, and many court appearances, the appeal is still pending with the Botswana courts.

## Discussion

### Using theory and research literature to interpret my story

As Johnson (2014) observes, theory and research literature and outcomes on bullying and mobbing make sense. For example, Gordon (2016) advises that when it comes to bullying, the best option is to look for the three most common components of bullying; these entail power imbalances, repetitive actions, and intentional actions and practices meant to harm the victims. In my case, all the three criteria specified by Gordon were met. Secondly, the perpetrators engaged in a series of repetitive behaviours and practices all aimed at harming my person, reputation and career. Given the intentional fabrications, I also believe that my story represents a clear witch-hunt against a target of bullying and is a classic case of unlawful discrimination in the workplace, still intended to harm both my person and my career prospects.

### Meaning in life amidst the bullying and mobbing

As I continued to wrestle with my integrity and deal with the humiliation of suspension, I was forced to confront some existential questions. As I noted before, I had studied psychology for a long time, and was a career academic teaching psychology. So, basically, my job and profession had become identical with “who I thought I was”. Therefore, when this part of my being was being questioned and threatened, I was forced to re-evaluate myself through questions such as: Why me? Why now? Is it possible to be anything other than a psychologist? What is the reason for my existence? What difference do I want to make for myself and others—and could I still make the same contribution outside my job and career? What should be my purpose and goals in life? How can I turn this crisis into an opportunity? These questions varied depending on the context and circumstances.

Researchers and practitioners have come to understand that searching for meaning in life, and making sense of situations—both positive and negative—are central to how people ensure that their lives seem organized, significant, purposeful and valuable (Steger, 2009; Steger, Frazier, Oisgi, & Kaler, 2006; Steger, Kashdan, Sullivan, & Lorentz, 2008; Taylor, 1983; Vanhooren, Leijssen, & Dezutter, 2016). Meaning in life has been conceptualized as “the extent to which people comprehend, make sense of, or see significance in their lives, accompanied by the degree to which they perceive themselves to have a purpose, mission, or over-arching aim in life” (Steger, 2009, p. 682). The presence of meaning has been defined as an individual’s perception of his or her life being significant, purposeful and valuable (Steger et al., 2006), such that people experience meaning when they comprehend the world, when they understand their place in it and can identify what they want to accomplish in life (Steger et al., 2008). Meaning in life concepts also converge with the concept of sense making, that is, the process through which people perceive their lives as meaningful even when they are not able to explain and understand their experiences (Proulx, Markman, & Lindberg, 2013). So, it is understandable that when a job or career is being threatened, a person can lose focus of their significance in life.

Several models have been proposed to understand how people organize, search, define, and find purpose and sense in their life circumstances, both negative and positive. Park, Riley, and Snyder (2012) and Joseph and Linley (2005) noted that traumatic events disrupt global meaning systems, and meaning-making coping helps to restore congruency between global meaning and appraisals of traumatic events by reducing the discrepancy between individual's appraised meanings and global beliefs and goals. Having noted this, meaning making refers to the processes in which people engage to reduce the discrepancy between appraised meaning and global beliefs and goals (Park, 2010; Park et al., 2012).

Meaning making appears particularly important in confronting highly stressful life experiences (Frankl, 1959; Park, 2010; Park et al., 2012; Taylor, 1983), as having a purpose and meaning in life are important in one’s chances of survival (Vanhooren et al., 2016). Others have noted that what matters, therefore, is not the meaning of life in general but rather the specific meaning of a person’s life at a given moment (Frankl, 1959, 1962; Machell, Kashdan, Short, & Nezlek, 2015). This awareness, knowledge and understanding is crucial for individuals dealing with traumatic experiences such as workplace mobbing and bullying.

Taylor (1983) proposed a theory of cognitive adaptation and suggested that the adjustment process centres on the following three themes: (1) a search for meaning in the experience; (2) an attempt to regain mastery over the event in particular and over life more generally; and (3) an effort to restore self-esteem through self-enhancing evaluations. Taylor’s model further suggests that successful adjustment depends, in a large part, on the ability to sustain and modify illusions that buffer not only against present threats but also against possible future threats. Taylor’s theory is used to describe how I coped with the bullying and mobbing.

### Coping with threatened job security

Having started by highlighting the importance of work for humanity, it is also important to discuss the consequences of loss of work or threatened job security. Jahoda (1981) warned of the consequences of loss of employment and called it psychologically destructive. While important, threatened job security in bullying situations has not yet received significant attention from researchers. Research has shown that because of the widespread value placed on work, being terminated from work can be traumatic (Spera, Buhrfeind, & Pennebaker, 1994) and can lead to mental illness, suicide, child abuse and hospital admissions (Brenner, 1976). Job insecurity has been defined as perceived powerlessness to maintain desired continuity in a threatened job situation (Greenhalgh & Rosenblatt, 1984). According to Ferrie (2001), job insecurity can be self-perceived or externally attributed, and self-perceived job insecurity is composed of individuals who report their jobs as insecure. While the severity of the threat to the work situation depends on the scope and importance of the potential loss, Greenhalgh and Rosenblatt (1984) have shown that the anticipation of job loss produces the same reaction as an anticipated death. They further explain that the scope of potential loss may depend on whether: (1) the anticipated loss is temporary or permanent; (2) the action causing the loss is layoff or firing; and (3) the change represents loss of the job itself or loss of job features. Related to Greenhalgh and Rosenblatt’s (1984) study, Ferrie (2001) posited that perceived job insecurity is considered a more potent stressor than an anticipated death. The literature has also shown that it is likely that individual differences moderate the relationship between experienced job insecurity and individuals’ reactions to it (DeFrank & Ivancevich, 1986; Greenhalgh & Rosenblatt, 1984), influencing how job loss or job insecurity harms psychological and physical health as well as influencing the coping strategies selected for dealing with the job loss (DeFrank & Ivancevich, 1986; Greenhalgh & Rosenblatt, 1984).

Researchers have also noted that the targets of bullying at work anticipate the workday with dread and a sense of impending doom. They go through the workplace in a state of high alert, in anticipation of the next attack. Privately, they are profoundly ashamed of being victimized and are confused at their apparent inability to fight back and protect themselves (Randall, 2001); this I also experienced.

My perpetrators knew that I had two small children—both going to private schools—and that I had a mortgage and many other financial commitments. They also knew that the job market in Botswana, especially for psychologists and academics, was tough. Furthermore, given the public nature of the allegations, even in a good market, finding another job could have been a tall order for me. Therefore, the threat of losing my job, which I loved dearly, indeed elicited the same reaction as an anticipated death, as Greenhalgh and Rosenblatt (1984) suggest.

Greenhalgh and Rosenblatt (1984) further explain that the sense of powerlessness is an important element of job insecurity, and has been suggested to be exacerbated if: (1) the organization had no strong norms of fairness; (2) the employee had no input into decisions and no right of appeal; and (3) superiors are seen as arbitrary in their evaluations and even capricious in their decisions affecting employees. This also depends on the employee’s beliefs about the organization’s standard operating procedures for dismissing employees. All these element of job insecurity were present at my place of work. Knowing that the head of an institution was colluding in efforts to frustrate, harass, torment and tear me down, and ultimately dismiss me from work, was scary—and left me feeling helpless and powerless.

Furthermore, when studying the letters and emails and preparing court documents, it became clear that there were major partialities and a lack of consistency in the application of the university’s procedures, rules and regulations. Therefore, when your employing institution is like mine, you are also likely to experience feelings of anger and rage about the lack of procedural fairness and even legal remedies. I also realized without policies and procedures to protect workers from perpetrators of workplace mobbing and mobbing, as long as you one is a manager or a supervisor, it is fully legal to bully others. Johnson (2014) advises that academic bullying will not stop until colleagues and administrators refuse to participate in mobbing; in my situation, it was difficult for people to refuse to participate because of the involvement of the head of the institution. This was very unfortunate because while some colleagues and administrators told me how unjust the situation was, they also expressed their challenges given the conflicted nature of the situation.

### Specific strategies employed to minimize the impact of bullying

#### Strategy 1: writing therapy

Through sharing experiences and coping ideas with other victims of workplace bullying, it became very clear to me that many victims want to be heard, and that in wanting the listener to understand their lived experiences, most questions asked to victims are commonly responded to in long, detailed and at times graphic narrations, making both ethnography and autoethnographies important research designs and approaches for understanding the lived experiences of victims and survivors of workplace bullying. I therefore decided to confront my experiences and feelings through journalling; journals which formed part of this autoethnography. This process was difficult at first because it was like I was reliving the experiences. While autoethnography may represent a conscious breaking away from formal academic writing, as suggested by Allison and Lawless (2011), in my experience the method has the following advantages: (1) it enhances cultural understanding of the self and others; (2) it facilitates transformation of the self and others through the process of doing, sharing and reading others’ and the self’s experiences (Chang, 2016); and (3) it offers a research method friendly to researchers and readers by allowing the researcher to locate their “self” as the subject for analysis and by narrating and interpreting events in a style that makes knowledge more accessible (Allison & Lawless, 2011; Chang, 2016).

Through journalling, I was better able to confront my “demons”. For example, prior to being a target of bullying and mobbing, I had suffered some setbacks in life—including incidents like the death of a mother, a grandmother, a grandfather, a brother and two close cousins—and I had convinced myself that I was trained to deal with psychological and social pain. I discovered that I was wrong. Having lived in the “bully lane”, I can certainly say that the pain of being bullied at work is among the most painful and traumatic experiences I have ever encountered, and that handling it is a difficult, long and tedious process. First, bullying and mobbing targeted my person as well as my integrity as both a human being and a professional. Secondly, it substantially affected my health. Thirdly, the experience almost bankrupted me. I also realized that the trauma was indeed also a function of the intensive fear and dread, explaining why bullying and mobbing are often called “psychological terror” (Leymann, 1996). The experience was also profoundly stigmatizing and embarrassing because the abuse, fictitious incidents and public humiliation became the main subject of different conversations across the university. As Westhues (2004) suggests, I felt like I was abhorrent, with no redeeming qualities outside the circle of acceptance and respectability, deserving only contempt. I was even more frustrated that I was not given a fair platform to defend my person. But somehow, putting all these feelings and fears on paper made me feel lighter, and helped me to think objectively of strategies to protect myself.

#### Strategy 2: sense making/meaning making

As I carried out research on the impact of bullying and mobbing, I was able to understand my experiences, at least intellectually. For example, studying research that discussed the outcomes of bullying and recognizing the commonalities between my experiences and those of others helped me to affirm that I was not necessarily weak or weird. Research also revealed to me that the trauma did not have to destroy my mental or physical health. For example, I came across a study of cancer patients where Taylor, Lichtman, and Wood (1984) revealed how the patients who were able to find meaning in their experience felt a sense of control, restored self-esteem, and adjusted better emotionally, compared to those who could not. I also read a lot, and watched documentaries on people who managed to overcome even worse circumstances. In this process I came across a book chronicling the experiences of an Auschwitz concentration camp inmate and survivor during World War II. In this book, the psychiatrist Viktor Frankl (1959) describes the psychotherapeutic method he used when he had to labour in four different concentration camps between 1942 and 1945. During this period, his parents, brother and pregnant wife died. Frankl (1959, 1962) suggests that while human beings cannot avoid human suffering, we can choose how to cope with it, find meaning in it, and move forward by identifying a purpose in life, and then immersively imagining a positive outcome and feeling positively about it. Therefore, through studying other people’s experiences, I was able to at least intellectually realize that being emotionally drained and physically sick was not necessarily a sign that I was a “weakling”.

#### Strategy 3: regaining mastery over the event

In an attempt to regain mastery over my life I discovered that staying engaged and active in the community improved my state of mind. For example, while serving suspension, I revisited my love of events and I organized conferences and workshops that brought stakeholders from different sectors of the Botswana economy together in a strategic and cohesive way to generate practical interventions that will assist the country to design implementable strategies to enable job creation, job expansion and job enhancement. One of the events became a national event that many stakeholders look forward to. I also continued to carry out research and publish my work. Accordingly, during the three years that I was suspended, I was able to work collaboratively with others and completed and published six academic publications (i.e., journal articles and book chapters with work in progress). These achievements helped me to find meaning and regain mastery of the suspension and other malicious accusations.

#### Strategy 4: self-enhancing evaluations

If you come from a culture like mine—a culture that is characterized by large power distance, respect for authority, strong uncertainty avoidance, collectivism, masculinity, short-term orientation and restraint (Pheko et al., 2017)—you are likely to engage in some form of self-doubt, self-stigma and self-blame when you are put in a position where you have to differ from authority. Through psychotherapy and writing therapy, I realized that for many months I just could not fathom or comprehend how a group of psychologists, university professors and seemingly sane-looking people could intentionally team up and unanimously agree to hurt, target, intimidate, humiliate, suppress, exclude, malign, discredit and intentionally fabricate stories about another human being. For some strange reason, I had naively thought that the perpetrators were somehow confused and I secretly blamed myself for their inability to understand me. Because of this “denial”, I spent months and a great deal of energy writing letters, negotiating meetings and raising my hand to try to explain myself. I went from the lowest office to the highest offices of the institution trying to explain how my works and I were misunderstood. I had wrongly convinced myself that if I could explain myself, to somebody, more eloquently, using different approaches such as meetings, letters and negotiation, then the negative actions and practices would cease. Well, I was wrong. I later noticed that my efforts only made matters worse as they gave the mob ammunition and justification to profile me as a “problem maker” who deserved to be summarily dismissed.

Therapy and support from colleagues assisted to me to realize that being a victim of mobbing and bullying did not make me a weak person. From reading Viktor Frankl’s book, and reflecting on the experiences of men like Nelson Mandela, who spent 27 years in prison, I was also able to reflect: “if human beings could walk through such traumatic events, I sure can survive these experiences”, which were, comparatively, a walk in the park. After this realization, and while still serving suspension, I decided wake up in the morning at the usual hour for work, dress up as if I were going to work, give myself targets and reward myself for reaching the targets. I also started going to church, where I learnt to rely on a source greater than myself. In church, I learnt that: “To everything there is a season, and a time to every purpose under the heaven” (Ecclesiastes 3:1). This empowered me to see my situation as a season that would definitely pass. I literally woke up in the morning and reminded myself: “this too shall pass”. In church I also discovered the power of true and sincere forgiveness, a lesson that miraculously relieved a lot of the psychosomatic and post-traumatic stress-related symptoms. Now, I sincerely believe that having gone through the bullying and mobbing experiences, I have become a better human being, able to relate better to myself and others around me.

### Implications for practice

Taylor (1983) proposed that the adjustment process centres on the following three themes: (1) a search for meaning in the experience (achieved by positive growth); (2) an attempt to regain mastery over the event in particular and over life more generally (achieved by changes in controllable aspects of life); and (3) an effort to restore self-esteem through self-enhancing evaluations (achieved through downward social comparison). The current study provides support for the three strategies as well as writing therapy/journalling. Past studies have also applied cognitive adaptation successfully. For example, the themes that emerged in Dibb and Kamalesh (2012)’s study on HIV-positive African women suggested that participants coped positively with their illness by positively interpreting their situation and making behavioural changes as well as using a variety of methods to rebuild self-esteem and create positive life meaning, offering support for cognitive adaptation theory. Nonetheless, others such as Aspinwall and Taylor (1992) have shown how different attributes of an individual may influence the coping process. For example, in the current study, writing therapy was highlighted as important part of cognitive appraisal of stressful life events. However, not everyone can read and/or write, meaning that those who wish to apply the strategies in their personal or professional settings need to be versatile in how they assist themselves or their clients to document their experiences. Given the efficacy of cognitive adaptation strategies, and the challenges highlighted above, I wish to propose cognitive adaptation training and coaching programmes designed for individuals who are undergoing stressful life events. The training and coaching programmes could be developed and implemented to educate individuals on how to apply the three strategies as well as educating the clients on the efficacy of the different strategies. The training coaching programmes should also accommodate a variety of demographic characteristics.

### Limitations of the study

While the importance of the current research has been mentioned, some limitations of this study should be noted. Similar to other qualitative studies, the main limitations that could be highlighted relate to the validity, reliability and generalizability of the study. While these limitations are acknowledged, for an autoethnographic study to be regarded as valid, the “story” should evoke feelings that the experience described is credible, realistic and believable (Ellis, 2004; Ellis et al., 2011). Still on validity, the readers could validate the stories by contrasting them with their own experiences. Reliability is also very important but it is assessed differently for autoethnographic studies. Reliability in this context means that the narrator’s credibility as juxtaposed with available “factual evidence” is essential (Ellis, 2004; Ellis et al., 2011). Reliability could also be judged in terms of whether the story helps readers to communicate with others, and/or whether the story offers a way of improving the lives of participants, readers and/or the author(s) (Ellis, 2004; Ellis et al., 2011). Generalizability is also assessed differently by readers seeking to determine whether a story speaks to them about their experience or about the lives of others they know (Ellis, 2004; Ellis & Bochner, 2000; Ellis et al., 2011). Lastly, while there are research questions that are best answered through quantitative research, research that is analytical, subjective, emotional, therapeutic and inclusive is also necessary (Ellis et al., 2011). Some have argued that such research allows authors to write through their pain (Ngunjiri et al., 2010), and encourages participants, researchers and readers to engage emotionally as well as cognitively (Liggins, Kearns, & Adams, 2013).

## Conclusions

Having gone through this experience, I can confirm that being bullied or mobbed at work can indeed be understood as the stressor to beat all stressors (Westhues, 2004). I also agree with those who compare it to the experience of being raped (Motin, 2009), divorced, losing a loved one through death (Mikkelsen & Einarsen, 2002) and going through a battle, water torture, nightmare or a noxious substance (Tracy et al., 2006). Furthermore, because bullying and academic mobbing are considered to be forms of discrimination equal to sexual harassment and racial discrimination, in stable and sane organizations, managers and supervisors must recognize that they have a moral and ethical obligation to protect employees from such hostile work environments. However, in chaotic organizations such as my employing organization, where protective policies are non existent, managers and supervisors seem to have legitimate power to bully others.

Hodson, Roscigno, and Lopez’s (2006) study further shows that when there is a disjuncture between organizational and relational factors, the extent of bullying is determined by underlying, context-specific aspects of power. The unfortunate assumption is that in cases of bullying and mobbing the target is the one who suffers; but research has revealed that workplace and academic bullying is devastating to all involved, including the perpetrators (Kircher et al., 2011; Rhodes, Pullen, Vickers, Clegg, & Pitsis, 2010).

Lastly, organizations should work tirelessly to design bully-free work environments. Every organization should come up with policies and structures to protect the victims and to eliminate chaos, which has been proven to be key in the abuse of power (Hodson et al., 2006). The literature and best practice overwhelmingly place the responsibility for intervening in the culture of bullying and academic mobbing on the administration, chairs, deans and/or president of universities, as it is their responsibility to make sure that there is a safe environment for all employees (Kircher et al., 2011), and, given the power imbalances involved in bullying situations, I concur. Accordingly, to fully confront perpetrators and protect victims, I recommend stand-alone, high-quality non-bullying policies (Wiedmer, 2011) which would identify all forms of bullying and academic mobbing and specify the consequences of bullying others.
